# Characteristics of Individuals With Advanced HIV Disease and Risk Factors for Mortality in a Contemporary Cohort in South Africa

**DOI:** 10.1097/QAI.0000000000003767

**Published:** 2026-01-01

**Authors:** Joshua Fieggen, Graeme Meintjes, Andrew Boulle, Jonathan Euvrard

**Affiliations:** a Department of Engineering Sciences, Institute of Biomedical Engineering, University of Oxford, Oxford, United Kingdom; b School of Public Health, Faculty of Health Sciences, University of Cape Town, Cape Town, South Africa; c Wellcome Centre for Infectious Diseases Research in Africa, Institute of Infectious Disease and Molecular Medicine, University of Cape Town, Cape Town, South Africa; d Department of Medicine, University of Cape Town, Cape Town, South Africa; e Blizard Institute, Faculty of Medicine and Dentistry, Queen Mary University of London, London, United Kingdom; f Centre for Integrated Data & Epidemiological Research, School of Public Health, University of Cape Town, Cape Town, South Africa; g Western Cape Government: Health, Cape Town, South Africa

**Keywords:** Advanced HIV Disease, HIV-related mortality, HIV, survival analysis

## Abstract

**Background:**

Advanced HIV disease is a major contributor to the slowing decline in HIV-related deaths globally. However, limited data exist on which individuals with advanced HIV are at highest risk of death.

**Methods:**

A retrospective cohort study was nested within a larger cohort of 13 primary care HIV treatment facilities in Khayelitsha, South Africa. All adults who had a CD4 count <200 cells/mm^3^ between January 1, 2017, and March 31, 2021, were enrolled. Descriptive statistics were calculated, and the cohort was then restricted to those who had linked vital status information. We evaluated risk factors for mortality using Kaplan–Meier curves, and univariable and multivariable Cox proportional hazards models.

**Results:**

Between 19% and 28% of the larger cohort (n = 72,102) were estimated to have advanced HIV disease at any point during the study period. Of these individuals, 20% were on treatment, 40% were disengaged from care, and 40% were treatment naive at enumeration. Overall mortality was 12%, with mortality highest in the first year (6.8%) after enumeration. There were 608, 371, and 370 deaths among those disengaged, on antiretroviral therapy (ART), and ART naive, respectively, representing 14%, 17%, and 8% of individuals in each group. More than a quarter of all participants were found to have current tuberculosis at enrollment into the cohort.

**Conclusions:**

ART-exposed individuals with advanced HIV disease contribute substantially to ongoing HIV-related mortality in South Africa. Improved adherence and retention strategies within HIV programs could reduce this mortality.

## Introduction

Global outcomes among people living with HIV (PLHIV) have improved substantially during the past 2 decades with approximately 60% reduction in HIV-related deaths from the peak of an estimated 1.7 million deaths in 2004 to an estimated 690,000 deaths in 2019.^1,2^ This progress is largely attributed to expanded access to antiretroviral therapy (ART) and the shift toward treating people earlier in the course of infection.^3,4^ The global health policy of “universal test and treat” and the UNAIDS 95-95-95 targets hope to extend this progress with the objective of ending the HIV epidemic by 2030.^5–7^

However, advanced HIV disease (AHD)—defined by the World Health Organization (WHO) in adults as an individual with a CD4 count less than 200 cells/mm^3^ or WHO clinical stage 3 or 4 disease—remains common and threatens to derail this progress. AHD is considered one of the most important drivers of the slowing decline in HIV-related deaths seen in recent years.^8^ AHD increases the risk of opportunistic infections such as tuberculosis and cryptococcal meningitis, with high associated mortality due to advanced immunosuppression.^9,10^ AHD is contributed to by late ART initiation, disengagement from ART care, suboptimal ART adherence, and poor immunologic responses to ART. This study aims to identify and describe risk factors for mortality in a contemporary cohort of individuals with AHD in South Africa.

## Methods

### Setting and Data Sources

Khayelitsha, Cape Town’s largest township, has high rates of unemployment, poverty, and areas of high population density with high rates of in- and out-migration from surrounding regions and provinces.^11,12^ Khayelitsha has an HIV program with 13 provincial and municipal public-sector ART clinics that treated >30,000 patients in 2015.^11^

The Khayelitsha cohort is a continuously recruiting open cohort of PLHIV presenting to ART care facilities within Khayelitsha.^12^ This cohort was described in detail by Stinson et al^12^ in 2016. The cohort consolidates hospital, laboratory, pharmacy, and electronic disease register data through a unique patient identifier with the linkage and anonymization of the data done through the Western Cape Provincial Health Data Centre (PHDC).^13^ For this study, a retrospective cohort study was nested within this overarching cohort.

### Routine Laboratory Testing and Changes in South African ART Guidelines

The Western Cape Consolidated Guidelines for HIV treatment include routine laboratory tests to monitor for complications of HIV and its drug treatment.^14^ These are outlined in addendum A1, Supplemental Digital Content, http://links.lww.com/QAI/C575 and include CD4 count testing at baseline/diagnosis, 12 months on ART, and on return to care after treatment interruption. Thereafter CD4 count testing is only repeated in the presence of AHD, virologic failure, or at the discretion of the treating clinician.

There has been a progressive expansion of the criteria for ART eligibility in South African guidelines, as detailed in addendum A2, Supplemental Digital Content, http://links.lww.com/QAI/C575.^12,15,16^ As of September 2016, South Africa implemented the current international guidance of universal testing and treatment, meaning ART is provided for all PLHIV regardless of CD4 count.^17,18^

### Inclusion Criteria and Data Collection

This study included all adults who attended provincial public-sector ART clinics in the Khayelitsha area and had at least 1 CD4 count <200 cells/mm^3^ between January 1, 2017, and March 31, 2021. Clinical stage 3 or 4 disease was not considered as an inclusion criterion because of missing data.

Enumeration was defined as the date of the first CD4 count <200 cells/mm^3^ in the study period. Enumeration viral load (VL) was defined as the most recent VL within 1 year before or 2 months after enumeration, with suppression considered any VL <100 copies/mL to account for variation across laboratory reference ranges.

Pre-deidentified routinely collected data were obtained through the PHDC. The data were then restricted as per the inclusion criteria. Where available, mortality data were ascertained by the PHDC through the National Population Registry (NPR) before deidentification. This was only possible for participants with linked National Identity numbers. Ascertainment of tuberculosis (TB) was done through the PHDC through triangulation of routinely collected health data (see addendum B, Supplemental Digital Content, http://links.lww.com/QAI/C575). Finally, 3 ART engagement categories were defined using linked pharmacy data. Participants who had collected ART within 90 days before their enumeration CD4 count were characterized as on ART, participants who had previously collected ART but not within the past 90 days were characterized as disengaged from care, and participants who had no record of ever previously receiving ART within the province were characterized as ART naive.

### Data Analysis

Descriptive statistics, chosen by data distribution, were used to summarize the data stratified by NPR linkage, vital status, and ART category. Missing data were coded as missing. Participants with NPR linkage were censored on their date of death or March 31, 2021, if still alive. The cohort was then restricted to those with NPR linkage. Kaplan–Meier curves were used to explore the time to death among NPR-linked participants stratified by various covariates.

Associations with mortality were evaluated using univariable and multivariable Cox proportional hazards models. When the proportional hazards assumption was tested for the overall multivariable model, it was noted that some features violated the assumption, particularly in the first 180 days after enumeration. Alternative modeling strategies such as flexible parametric models,^19^ time transformations, step functions, and stratified models were considered. However, given the causal limitations of this study, a pragmatic decision was made to persist with the Cox modeling acknowledging its limitations. Because ART exposure was the most important violation, stratified models were also constructed. Owing to colinearity between enumeration VL and CD4 count, the 2 covariates were not modeled together. Finally, to evaluate the impact of ART category thresholds, a sensitivity analysis reclassifying all individuals who collected their first ART within 180 days as ART naive was performed (see addendum B, Supplemental Digital Content, http://links.lww.com/QAI/C575). All data were analyzed using *R Studio*.^20^ The definitions of terms are given in addendum C, Supplemental Digital Content, http://links.lww.com/QAI/C575.

### Ethics Statement

This study was approved by the University of Cape Town Human Research Ethics Committee with reference 810/2020 as a substudy of an ongoing open cohort study (HREC 395/2005). The STROBE checklist for this study is presented in addendum D, Supplemental Digital Content, http://links.lww.com/QAI/C575.

## Results

### Cohort Characteristics

**Figure 1:**
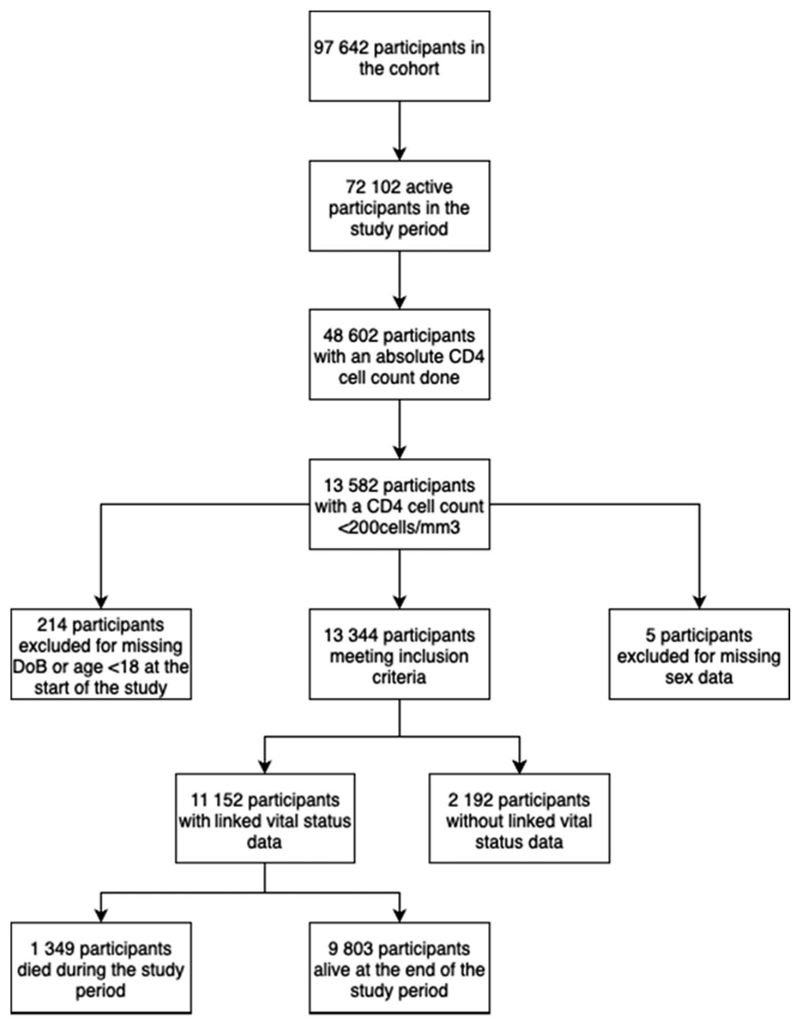
Flowchart of participant selection and outcome in the study cohort. This flowchart illustrates the selection process and outcomes of participants included in the study cohort. The cohort initially comprised 97,642 participants, from whom 72,102 were identified as active participants during the study period. Among these, 48,602 participants had a CD4 count measured. The analysis focused on 13,582 participants who had an absolute CD4 count <200 cells/mm^3^, indicating advanced HIV disease. After applying exclusion criteria, 214 participants were excluded because of missing date of birth or being younger than 18 years of age at the start of the study. An additional 5 participants were excluded because of missing sex data. This left 13,344 participants who met the inclusion criteria for the study. These 13,344 participants were then categorized based on whether their vital data were linked to the NPR. Of these, 11,152 participants had linked vital data, while 2192 participants did not. The outcome for those with linked vital data showed that 1349 participants died during the study period, while 9803 survived.

In total, 97,624 participants had at least 1 HIV-related investigation at 1 of the 13 ART clinics in the Khayelitsha Cohort (Fig. 1). Of these participants, 72,102 were active within the study period, 48,602 (67%) of whom had at least 1 CD4 count done within the study period. In total, 13,582 participants had at least 1 CD4 count <200 cells/mm^3^. Thus, 19% of all active participants and 28% of all participants who had a CD4 count had AHD.

Next, 219 participants were excluded for missing demographic data. Of the 13,344 participants meeting inclusion criteria, 11,152 (84%) had linked vital status data (Table 1). Unlinked participants were noted to be more likely to be male and were older (see addendum E, Supplemental Digital Content, http://links.lww.com/QAI/C575). The median follow-up time was 896 days [interquartile range (IQR): 490–1272]. Male participants constituted 43% of the cohort and the median age was 35.8 years (IQR: 30.3–42.7). Median enumeration CD4 count was 111 cells/mm^3^ (IQR: 57–158) and modal CD4 category was 100–199 cells/mm^3^ (55%). An enumeration VL was identified for 30% of participants, a third of whom (32%) were suppressed. Only 16% of the cohort had their enumeration CD4 count taken in a hospital facility. However, 31% of individuals who subsequently died and 24% of people on ART at enumeration had their enrollment CD4 count taken in a hospital.

### ART Engagement

At enumeration, 2631 (20%) participants in the cohort were on ART, 5280 (40%) participants were previously on but currently disengaged from ART, and the remaining 5433 (40%) participants had no record of previous ART exposure in the province (ART naive). Among individuals who were disengaged from care at enumeration, 4853 (92%) reinitiated ART, with a median time from index CD4 count to reinitiation of 7 days (IQR 0–25) (Table 1; see Addendum F, Supplemental Digital Content, http://links.lww.com/QAI/C575). Of these, 3495 (72%) went on to receive at least 1 ART refill ≥180 days after reinitiation. Of the 5433 participants who were ART naive at enumeration, 95% were subsequently initiated on ART with a median time to initiation of 11 days (IQR: 0–28). Of the 2631 participants on ART at enumeration, 512 (19%) had collected their first ART within a year before enumeration with a median time on ART at enumeration of 97 days (IQR: 14–237 days) while the remaining 2119 participants had first collected ART more than a year before enumeration (median 2163 days; IQR: 1245–3202). In total, 79% of participants on ART at enumeration had enumeration VLs, 44% of which were suppressed.

### Comorbidities

A third of all participants had evidence of TB >2 months before enumeration (previous TB) (Table 1). Previous TB was most common among participants on ART (55%) or disengaged (47%) and least prevalent among ART-naive participants (8.2%). A quarter (26%) of all participants had evidence of current TB at enumeration. Current TB was found in 24% and 31% of participants who were disengaged from care and ART naive, respectively, as compared with 16% of participants on ART at enumeration. There was microbiologic evidence of *Cryptococcus neoformans* in CSF or serum/plasma within 2 months before or after enumeration for 1.6% of participants.

Almost all (99.8%) participants enumerated with a CD4 count <100cells/mm^3^ had a reflex cryptococcal antigen test done on their blood, 3.4% of which were positive. Of the 199 of these participants, 64 had a subsequent CSF cryptococcal investigation within 30 days and 135 (67.8%) were prescribed fluconazole (at any dose) within 90 days of the positive result. Of the whole AHD cohort, 5857 (43.9%) and 9277 (69.5%) of participants were prescribed co-trimoxazole within 90 days of enumeration and at any point during the study, respectively.

### Mortality Distribution by ART Exposure

Of the 11,152 NPR-linked participants, 1349 (12%) died within the study period, 371 (28%), 608 (45%), and 370 (27%) of whom were on ART, disengaged, and ART-naive categories, respectively (Table 1). One-year mortality was 6.8%, representing 52% of the 1243 deaths among participants eligible to be followed for at least a year. Of the 3871 participants enumerated with a CD4 count <100 cells/mm^3^ and able to be in the study for at least a year, 431 (11%) died within 1 year, representing 55% of the 777 deaths in this group. There was a positively skewed distribution of time to death from enumeration across all enumeration ART statuses (see addendum G, Supplemental Digital Content, http://links.lww.com/QAI/C575) with an overall median time to death of 281 days (IQR: 59–681).

### Mortality Risk by ART Exposure

**Figure 2:**
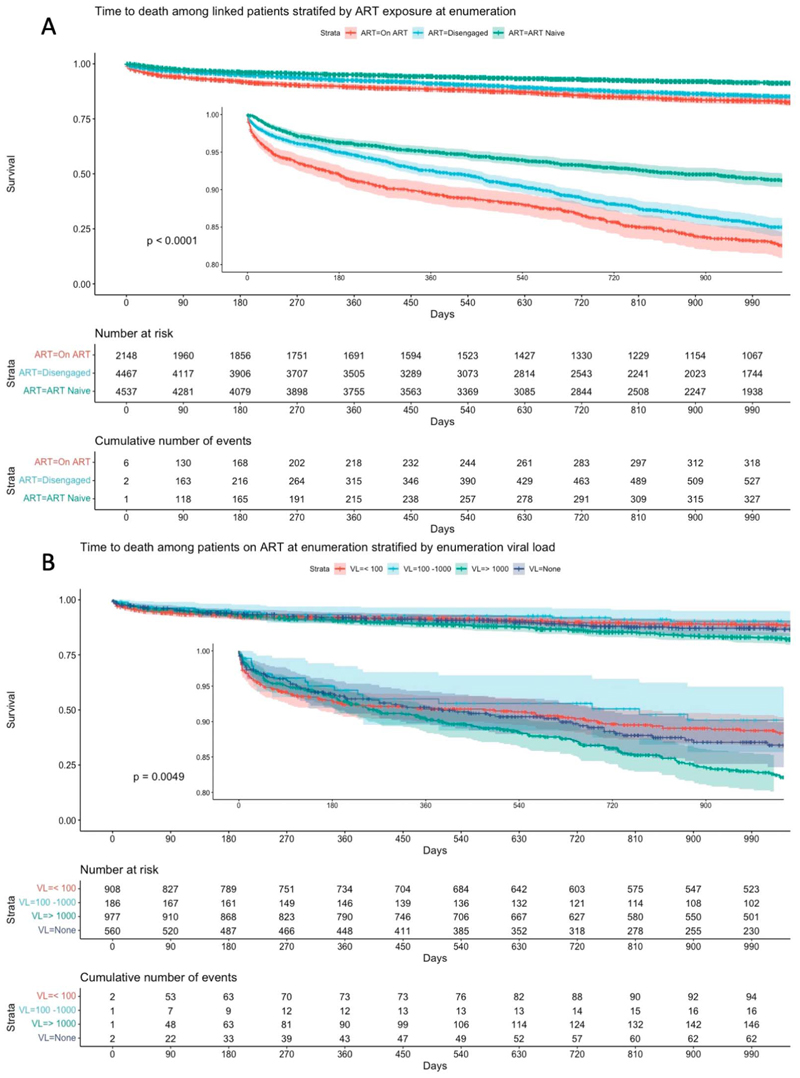
Kaplan–Meier survival curves for time to death stratified by ART status and viral load at enumeration. This figure presents Kaplan–Meier survival curves illustrating the time to death among participants with AHD, with data censored at 1000 days of follow-up. Panel A shows the survival curves stratified by ART status at the time of enumeration: participants actively on ART (red), disengaged from ART (blue), and ART naive (green). The inset highlights the initial 180 days, where the difference in survival between the groups is most pronounced. The log-rank test indicates a significant difference in survival between the groups (*P* < 0.0001). Panel B displays survival curves among participants on ART, further stratified by VL at enumeration: VL < 100 copies/mL (red), VL between 100 and 1000 copies/mL (blue), VL >1000 copies/mL (green), and those with no available viral load data (purple). The inset again focuses on the first 180 days of follow-up, emphasizing the early differences in survival based on viral load. The log-rank test shows a significant difference in survival across these strata (*P* = 0.0049). For both panels, the tables beneath the curves detail the number of participants at risk and the cumulative number of events (deaths) at various time points during the follow-up period.

Of the 2148 linked participants on ART at enumeration, 17% died during the study period, while 14% and 8% of disengaged and treatment-naive participants, respectively, died (Table 1). Prior treatment (either on ART or disengaged at enumeration) was more common among participants who died (73%) compared with participants who survived (57%). Being disengaged from care at enumeration carried an unadjusted survival benefit of 15% (unadjusted hazard ratio (HR) 0.85 [95% confidence interval (CI) 0.75 to 0.92]) and being ART naive reduced the hazard of death by half (HR 0.48 [95% CI: 0.42 to 0.56]) compared with being on ART at enumeration. This is highlighted in a Kaplan–Meier analysis in Figure 2.

### Additional Univariable Associations

Male sex and increasing age were univariable demographic predictors of mortality (Table 2). An unsuppressed VL was a predictor of mortality among participants on ART at enumeration (HR: 1.51 [95% CI: 1.19 to 1.92]) and a lower enumeration CD4 count was associated with mortality across all ART groups. Current TB and previous TB had unadjusted HRs for mortality of 1.88 (95% CI: 1.68 to 2.10) and 2.00 (95% CI: 1.80 to 2.23), respectively.

### Multivariable Analysis

Overall, being disengaged from care before enumeration was associated with an adjusted 18% (adjusted hazard ratio (aHR) 0.82 [95% CI: 0.72 to 0.93]) lower hazard of mortality, while being ART naive was associated with half the relative hazard of death (aHR 0.57 [95% CI: 0.49 to 0.67]) compared with those on ART (Table 2). Sex had no adjusted impact on survival. The lowest mortality was in the 25–35 years age category, while being older than 45 years was associated with the highest mortality. Enumeration CD4 count categories of 51–100 cells/mm^3^ and 0–50 cells/mm^3^ increased the adjusted hazard of mortality by 62% and 191%, respectively, compared with those with a CD4 count >100 cells/mm^3^. Previous TB had an aHR of 1.47 (95% CI: 1.37 to 1.59). Within the stratified models, male sex was a predictor of mortality among ART-naive individuals (aHR 1.30, 95% CI: 1.13 to 1.48) and VL >1000 copies/mL was a predictor of mortality among PLHIV on ART at enumeration (aHR 1.56 95% CI: 1.34 to 1.81).

### Sensitivity Analysis

Changing the definition of ART naive to include those who first collected ART within 3 months (90 days) before enrollment in the study increased the size of this group by 252 individuals. Repeated Kaplan–Meier curves showed minimal impact of this reclassification of the trajectories of the 3 groups (see addendum H, Supplemental Digital Content, http://links.lww.com/QAI/C575).

## Discussion

This study highlights the ongoing burden of AHD in South Africa with approximately a quarter of PLHIV in Khayelitsha who had contact with health services found to have advanced disease for the 4-year study period. The overall mortality during the study period was 12% with 52% of deaths occurring within the first year after enumeration. ART exposure was strongly associated with mortality. The findings emphasize the increasing importance of treatment failure and disengagement from care in driving ongoing HIV-related mortality^21,22^: 79% of treatment-experienced PLHIV in the cohort were either disengaged from care or on ART but viremic at enumeration. Considering most of these individuals can be presumed to have unsuppressed VLs, this may have significant implications for both HIV transmission^23–25^ and mortality.^26^ Adjusted risk factors for mortality were largely consistent across all ART categories with older age, lower CD4 counts, and previous TB at enumeration emerging as important independent predictors.

### AHD Prevalence

The 19% of the active HIV cohort who had a CD4 count <200 cells/mm^3^ is likely an underestimate of true AHD prevalence in Khayelitsha because the dropping of universal routine annual CD4 counts means some participants with AHD would have been missed, while the high proportion (28%) of those who had CD4 counts done with AHD is likely an overestimate because sick participants are both more likely to have AHD and have a CD4 count done. Taking a midpoint estimate, approximately a quarter of participants accessing HIV care in Khayelitsha within the study period had AHD. This is higher than the 16% AHD prevalence found across 4 similarly high-burden lower- and middle-income countries in 2016,^27^ but lower than the 34% prevalence described in a recent study from Kenya.^28^

### Mortality

Within this cohort, 1-year mortality was 6.8% overall and 11% among participants enumerated with a CD4 count of <100 cells/mm^3^. This is similar to the 13% mortality in participants with CD4 counts <100 cells/mm^3^ who died within 48 weeks in the REALITY trial,^29^ and underscores the ongoing high mortality associated with AHD. Deaths also typically occurred early after enrollment into the cohort. These findings emphasize the importance of the WHO bundle of care for participants with AHD on entry or re-entry into care.^8,30^ Low enumeration CD4 count and increasing age were consistent predictors of mortality. These 2 risk factors have been found to be predictors in other settings.^10,31,32^

### ART

Most (60%) of this AHD cohort were treatment experienced, two-thirds of whom were disengaged at enumeration, emphasizing that many participants who re-present to care do so with AHD.^33^ The adjusted hazard of mortality was lowest among ART-naive participants followed by participants disengaged from care at enumeration. This paradoxical association between ART experience and increasing mortality might be influenced by several factors. First, enrollment in this cohort required a defined HIV episode that inherently relied on contact with the health services. Therefore, the participants most likely missing from this cohort are those who die with AHD without ever engaging with ART services. This may have falsely lowered the risk of mortality among ART-naive participants. There are also few high-quality trials on effective interventions for addressing the key issue of adherence in this population.^34^ Furthermore, because all individuals who are ART naive are still eligible to receive CD4 counts irrespective of their clinical presentation, it is likely that more asymptomatic individuals with incidental low CD4 counts were included in this group, compared with those re-presenting to care after an interruption, often because of symptoms.

In addition, the high rates of viremia among those on ART specifically are likely important. A VL >1000 copies/mL at enumeration was an independent risk factor for mortality, in keeping with contemporary literature.^26^ In total, 44% of participants on ART who had an enumeration VL on record were virologically suppressed. This suggests that many participants who have AHD, despite being on ART, have complex adherence challenges that result in viremia and associated mortality, or may have recently restarted ART after a period of disengagement and subsequent return to care because of morbidity. In addition, across resource-limited settings, there are delays in switching to second-line ART in the presence of drug resistance, even when routine VL monitoring is available^35^ although the importance of this within this cohort was not investigated within this study. Next, the higher median age of those on ART (38.1 years) compared with those disengaged (36.0 years) and ART naive (34.5 years) may suggest a longer duration because of a first low CD4 count and/or HIV diagnosis, which could contribute to the cumulative risk of opportunistic infections and other complications. Nadir CD4 (lowest ever CD4) specifically has been shown to be strongly predictive of long-term mortality among participants who have been on ART for at least 1 year.^36^ Finally, clinically unwell individuals on ART are more likely than their well counterparts to have a CD4 count done and, therefore, are disproportionately likely to be included in this cohort. This is underscored by the high rate of enumeration in a hospital setting and the high acute mortality risk among those on ART.

Disengagement from care at enumeration was common (40%) within the cohort and the association with increased mortality compared with the ART-naive group persisted despite controlling for important covariates. This finding underscores the challenge disengagement from care presents to HIV programs globally^37^ and highlights the importance of conceptualizing the HIV care cascade as cyclical rather than linear and planning services around the “revolving door” of disengaging and re-engaging with care to improve retention and reduce deaths.^38^ It also emphasizes the need to consider alternative models of ART delivery such as nonhealth facility-based care^39,40^ and multimonth dispensing.^41,42^ There is contemporary evidence that many of these interventions may be more cost-effective than initially estimated.^43^

Despite approximately a third of ART-naive PLHIV entering care with current TB, this ART-naive group had the lowest mortality of 8.2%. However, this should be interpreted within the aforementioned context that this may be an underrepresentation of mortality within this population. It is notable within the analysis of the ART-naive group that male participants had a higher unadjusted and adjusted risk for mortality. This is in keeping with historical analyses of ART-naive cohorts^44^ and may be linked to the known propensity for male participants to disengage from care,^45,46^ further underscoring the importance of sustained retention on treatment for reducing AHD-related mortality.

### Comorbidities

There was a high prevalence of current TB within the cohort. Current TB was particularly common among ART-naive (31%) and previously disengaged (24%) participants compared with participants on ART (16%). This suggests that TB is important in driving participants with AHD to seek or return to care, and emphasizes the need to actively rule out TB at presentation.^30^ The high overall prevalence of current TB within the cohort is likely because of a combination of the known very high risk of TB in AHD^47^ (especially within sub-Saharan Africa), the propensity for TB to lead clinicians to do CD4 count testing, and the direct lymphopenic effect of TB.^48^

Previous TB was shown to confer an approximately 50% adjusted increase in the risk of death across all ART categories. This is likely attributable to PLHIV, particularly those with low nadir CD4 counts, being at particularly high risk for recurrent TB.^49–52^ More than a third (37%) of participants who subsequently died had current TB at the time of enumeration with the risk of associated mortality being greatest among ART-naive participants. This is in keeping with recent literature that suggests that TB remains one of the most important causes of death in people with AHD,^8,29,53,54^ with TB accounting for one-third of all HIV-related deaths worldwide in 2018.^55^

Reflex cryptococcal antigen screening on samples with CD4 counts <100 cells/mm^3^ was introduced in 2016.^56^ The importance of this is highlighted in the cryptococcal anti-genemia rate of approximately 4% found in this study. However, the subsequent CSF investigation (32% within 30 days) and prescription of fluconazole (68% within 90 days) highlight potential gaps in the management of these individuals. In addition, the relatively low rates of cotrimoxazole prescription (44% within 90 days of the index CD4 count across the whole cohort) in the context of guidance to initiate and continue prophylaxis in all individuals with a CD4 count <200 cells/mm^3 57^ are of additional potential concern.

### Limitations

Whereas this study initially sought to explore the associations between prior ART exposure, interruptions, and mortality, an important limitation is that because annual CD4 counts are no longer routinely done as per the 2015 changes in the local guidelines, there are multiple potential biases in AHD ascertainment. For example, there could be a propensity to under-ascertain AHD in those already in care. In addition, to be included in the cohort, a participant had to have attended an ART clinic in Khayelitsha. This would have led to underascertainment of the number of ART-naive participants diagnosed at a hospital level who, because of death, outward migration, or disengagement from care, were never linked to primary care. In addition, individuals who collect ART in other provinces would not be noted in this data set. This carries significant implications, notably including that PLHIV collecting ART from other provinces could incorrectly be classified as disengaged or ART naive. The analysis is also limited by a lack of access to cause-of-death information,^58^ which notably limits a full understanding of the impact of OIs such as TB on this population. Finally, engagement status is looked at cross-sectionally and is complexly associated with morbidity, and care trajectories thus cannot be interpreted as causally associated with mortality.

### Future Research

This study demonstrates that there remain many PLHIV with AHD who have disengaged from care. Although risk factors for disengagement are well documented,^11^ it will be important to identify interventions to promote sustained retention after ART reinitiation. This study has also highlighted the complex longitudinal relationship between different patterns of care engagement and potential risk factors for mortality. Evaluating the impact of newer drug regimens such as tenofovir–lamivudine–dolutegravir, which may modify patterns of engagement through improved tolerability, is important for future research.

## Conclusions

It has been noted that as HIV epidemics develop, individuals disengaged from or failing ART contribute an increasing proportion of HIV-related deaths and transmissions.^59^ This study has highlighted the relevance of this shift in advanced HIV disease in South Africa and the substantial contribution of this to ongoing HIV-related mortality in South Africa. This has important implications for the HIV program across lower- and middle-income countries and emphasizes the need to consider both interventions that improve engagement with care, such as 6-monthly dispensing, as well as interventions specifically aimed at reducing the number of people entering care with a low CD4 count such as targeted HIV testing of groups in the population at high risk of having AHD.

## Supplementary Material

Supplemental Digital Content

## Figures and Tables

**Table 1:** Characteristics of Adults With Advanced HIV by Linkage, Mortality Status, and ART Category at Enumeration

Linkage and Mortality Status							
*Characteristic*	Linked		Unlinked	ART Category at Enumeration			Overall
	Survived	Died		On ART	Disengaged	ART Naive	
N (% of overall)	9803 (73.5)	1349 (10.1)	2192 (16.4)	2631 (19.7)	5280 (39.6)	5433 (40.7)	13,344 (100)
NPR linkage (linked) (%)	9803 (100)	1349 (100)	0 (0.0)	2148 (81.6)	4467 (84.6)	4537 (83.5)	11,152 (83.6)
Mortality among NPR linked (died) (%)	0 (0.0)	1349 (100)	—	371 (17.3)	608 (13.6)	370 (8.2)	1349 (12.1)
Enumerated in hospital (%)	1248 (12.7)	422 (31.3)	393 (17.9)	633 (24.1)	855 (16.2)	575 (10.6)	2063 (15.5)
Sex (male) (%)	4069 (41.5)	635 (47.1)	1024 (46.7)	1107 (42.1)	1988 (37.7)	2633 (48.5)	5728 (42.9)
Age categories							
18–25 yrs (%)	805 (8.2)	90 (6.7)	136 (6.2)	149 (5.7)	356 (6.7)	526 (9.7)	1031 (7.7)
25–35 yrs (%)	3976 (40.6)	425 (31.5)	753 (34.4)	822 (31.2)	2028 (38.4)	2304 (42.4)	5154 (38.6)
35–45 yrs (%)	3350 (34.2)	481 (35.7)	806 (36.8)	961 (36.5)	1951 (37.0)	1725 (31.8)	4637 (34.7)
>45 yrs (%)	1672 (17.1)	353 (26.2)	497 (22.7)	699 (26.6)	945 (17.9)	878 (16.2)	2522 (18.9)
Age (median [IQR])	35.3 [30.0, 41.9]	37.7 [32.0, 45.4]	37.2 [31.5, 44.4]	38.1 [32.3, 45.5]	36.0 [30.7, 42.4]	34.5 [29.3, 41.5]	35.8 [30.3, 42.7]
Enumeration CD4 (median [IQR])	117 [64, 161]	69 [30, 128]	107 [56, 157]	126 [73, 166]	106 [52, 155]	108 [56, 156]	111 [57, 158]
Enumeration CD4 categorical (%)							
101–199	5692 (58.1)	500 (37.1)	1176 (53.6)	1659 (63.1)	2784 (52.7)	2925 (53.8)	7368 (55.2)
51–100	2225 (22.7)	328 (24.3)	520 (23.7)	553 (21.0)	1235 (23.4)	1285 (23.7)	3073 (23.0)
0–50	1886 (19.2)	521 (38.6)	496 (22.6)	419 (15.9)	1261 (23.9)	1223 (22.5)	2903 (21.8)
Time in cohort* (median [IQR])	950.0 [568.0, 1294.0]	281.0 [59.0, 681.0]	973.5 [578.0, 1308.0]	1027.0 [490.0, 1371.5]	845.5 [447.0, 1220.0]	898.0 [527.0, 1267.0]	895.5 [489.8, 1272.0]

Viral load at enumeration† (%)							
<100	947 (9.7)	153 (11.3)	211 (9.6)	908 (34.5)	328 (6.2)	75 (1.4)^	1311 (9.8)
100–1000	318 (3.2)	47 (3.5)	66 (3.0)	186 (7.1)	177 (3.4)	68 (1.3)	431 (3.2)
>1000	1493 (15.2)	362 (26.8)	437 (20.0)	977 (37.1)	1009 (19.1)	306 (5.6)	2292 (17.2)
Not done	7045 (71.9)	787 (58.3)	1478 (67.4)	560 (21.3)	3766 (71.3)	4984 (91.7)	9310 (69.8)
ART (%)							
On ART	1777 (18.1)	371 (27.5)	483 (22.0)	2631 (100.0)	0 (0.0)	0 (0.0)	2631 (19.7)
Disengaged	3859 (39.4)	608 (45.1)	813 (37.1)	0 (0.0)	5280 (100.0)	0 (0.0)	5280 (39.6)
ART naive	4167 (42.5)	370 (27.4)	896 (40.9)	0 (0.0)	0 (0.0)	5433 (100.0)	5433 (40.7)
ART naive + subsequently initiated (%)	4026 (96.6)	325 (87.8)	833 (93.0)	—	—	5184 (95.4)	5184 (95.4)
Time‡ to initiation (median [IQR])	10 [0, 28]	15 [5, 31]	14 [2, 28]	—	—	11 [0, 28]	11 [0, 28]
Disengaged + Reinitiated (%)	3617 (93.7)	500 (82.2)	736 (90.5)	—	4853 (91.9)	—	4853 (91.9)
Time‡ to Reinitiation (median[IQR])	6.0 [0.0, 25.0]	8.0 [1.0, 27.0]	5.0 [0.0, 21.0]	—	7.0 [0.0, 25.0]	—	7.0 [0.0, 25.0]
Tuberculosis (TB)							
Current§ Tb (%)	2295 (23.4)	494 (36.6)	611 (27.9)	425 (16.2)	1278 (24.2)	1697 (31.2)	3400 (25.5)
Previous ∥ Tb (%)	2902 (29.6)	661 (49.0)	831 (37.9)	1441 (54.8)	2505 (47.4)	448 (8.2)	4394 (32.9)
Incident¶ Tb (%)	1533 (15.6)	328 (24.3)	351 (16.0)	451 (17.1)	1033 (19.6)	728 (13.4)	2212 (16.6)
Cryptococcosis							
Current§ Cryptococcosis (%)	135 (1.4)	50 (3.7)	40 (1.8)	48 (1.8)	92 (1.7)	85 (1.6)	225 (1.7)
Previous ∥ Cryptococcosis (%)	75 (0.8)	17 (1.3)	27 (1.2)	63 (2.4)	52 (1.0)	4 (0.1)	119 (0.9)
Incident¶ Cryptococcosis (%)	126 (1.3)	50 (3.7)	36 (1.6)	50 (1.9)	103 (2.0)	59 (1.1)	212 (1.6)

This table provides an overview of the demographic and clinical characteristics of 13,344 adults with advanced HIV in Khayelitsha. The table is stratified by participants who were linked to the national population registry with an identity number on whom vital status is ascertainable versus those who were unlinked (with no vital status information), as well as between those who survived or died among the linked group. In addition, participants are categorized based on their ART status at enrollment (enumeration) into the cohort: those currently on ART, those who had disengaged from ART, and those who were ART naive at the time of enrollment. Categorical variables are presented with corresponding percentages. Numerical variables are summarized using median values and IQR.

* Time in cohort denotes time in days from enumeration to censoring (either death or March 21, 2021).

† Individuals classified as ART naive are assumed to have suppressed within 2 months after enumeration or to have transferred from another province where they were collecting ART.

‡ Time in days from enumeration to first (subsequent) ART collection.

§ Evidence of comorbid condition within 2 months before or 2 months after enumeration.

∥ Evidence of comorbid condition >2 months before enumeration.

¶ Evidence of comorbid condition >2 months after enumeration.

**Table 2:** Univariable and Multivariable Cox Proportional Hazards Models for Mortality Risk Among Participants With HIV Stratified by ART Status

	Univariable			Overall		Univariable			On ART 1		On ART 2		Univariable			Disengaged		Univariable			ART Naive	
	N	HR*	95% CI*	aHR*	95% CI*	N	HR*	95% CI*	aHR*	95% CI*	aHR*	95% CI*	N	HR*	95% CI*	aHR*	95% CI*	N	HR*	95% CI*	aHR*	95% CI*
**Age**	11,152					2148							4467					4537				
25–35		—	—	—	—		—	—	—	—	—	—		—	—	—	—		—	—	—	—
18–25		1.03	0.82 to 1.29	1.20	0.95 to 1.50		0.68	0.38 to 1.20	0.69	0.39 to 1.23	0.70	0.39 to 1.25		1.36	0.99 to 1.86	1.54	1.12 to 2.12		1.02	0.68 to 1.53	1.21	0.80 to 1.83
35–45		1.36	1.19 to 1.55	1.20	1.05 to 1.37		1.09	0.85 to 1.41	1.08	0.83 to 1.39	1.07	0.83 to 1.39		1.23	1.02 to 1.49	1.10	0.90 to 1.34		1.64	1.28 to 2.11	1.52	1.18 to 1.96
<45		1.94	1.68 to 2.23	1.75	1.51 to 2.03		1.49	1.15 to 1.93	1.58	1.21 to 2.07	1.52	1.16 to 1.99		1.82	1.47 to 2.25	1.68	1.34 to 2.11		2.30	1.75 to 3.02	2.15	1.63 to 2.83
**ART**	11,152																					
On ART		—	—	—	—																	
Disengaged		0.85	0.75 to 0.97	0.82	0.72 to 0.93																	
ART naive		0.48	0.42 to 0.56	0.57	0.49 to 0.67																	
Sex (male)	11,152	1.22	1.09, 1.36	1.04	0.93 to 1.16	2148	1.14	0.93 to 1.39	0.96	0.77 to 1.19	1.03	0.83 to 1.27	4467	1.24	1.06 to 1.46	0.97	0.82 to 1.16	4537	1.47	1.19 to 1.80	1.28	1.04 to 1.58
CD4	11,152					2148							4467					4537				
101–199		—	—	—	—		—	—	—	—				—	—	—	—		—	—	—	—
51–100		1.60	1.39 to 1.84	1.62	1.41 to 1.86		1.83	1.42 to 2.34	1.81	1.41 to 2.33				1.74	1.40 to 2.17	1.71	1.38 to 2.13		1.38	1.05 to 1.80	1.35	1.03 to 1.77
0–50		2.94	2.60 to 3.33	2.91	2.57 to 3.30		2.67	2.09 to 3.40	2.66	2.08, 3.41				3.58	2.97 to 4.31	3.41	2.82 to 4.11		2.61	2.06 to 3.29	2.43	1.92 to 3.08
Previous TB	11,152	2.00	1.80 to 2.23	1.46	1.29 to 1.66	2148	1.58	1.27 to 1.97	1.45	1.16, 1.80	1.50	1.20, 1.88	4467	1.75	1.49 to 2.06	1.50	1.27 to 1.77	4537	1.65	1.21 to 2.26	1.48	1.08 to 2.02
Incident TB	11,152	1.34	1.19 to 1.52			2148	1.18	0.93 to 1.51					4467	1.29	1.08 to 1.54			4537	1.29	0.99 to 1.69		
Current TB	11,152	1.88	1.68 to 2.10			2148	2.21	1.75 to 2.79					4467	1.91	1.62 to 2.25			4537	2.31	1.88 to 2.84		
Previous cryptococcosis	11,152	1.40	0.87 to 2.25	0.88	0.54 to 1.42	2148	0.97	0.50 to 1.88	0.81	0.42 to 1.57	0.86	0.44, 1.66	4467	1.36	0.68 to 2.73	1.02	0.51 to 2.06					
Viral load at enumeration	11,152					2148							4467									
>100		—	—				—	—			—	—		—	—							
100–1000		0.97	0.70 to 1.35				0.95	0.59 to 1.53			1.00	0.62 to 1.61		1.08	0.63 to 1.86							
<1000		1.53	1.27 to 1.85				1.51	1.19 to 1.92			1.54	1.21 to 1.96		1.68	1.16 to 2.41							
None		0.78	0.65 to 0.93				1.11	0.82 to 1.50			1.27	0.93 to 1.73		1.10	0.78 to 1.55							
Enumerated in hospital	11,152	3.11	2.77 to 3.49			2148	3.65	2.97 to 4.48					4467	2.73	2.29 to 3.25			4537	2.35	1.80 to 3.05		

* HR, hazard ratio; CI, confidence interval.

This table presents the results of univariable and multivariable Cox proportional hazards models, assessing the impact of selected HIV-related covariates on mortality risk. The “Univariable” columns display the unadjusted hazard ratios (HR) for each risk factor within the respective (sub)cohorts. The “Overall” column reports the aHR for the entire cohort, while the “On ART 1”, “On ART 2,” “Disengaged”, and “ART Naive” columns represent aHRs in subgroups of participants: those on ART, those disengaged from ART, and those ART naive at the time of enrollment in the AHD cohort.

Key covariates include age, ART status, sex, CD4 count, history of TB or cryptococcosis, and viral load at enumeration. Owing to collinearity between CD4 count and viral load, 2 separate models were constructed (ART 1 and ART 2) with either CD4 count or viral load at enrollment. The estimated hazard ratios should be interpreted as weighted averages during the entire follow-up period. Notably, for many covariates, the risk of mortality is highest shortly after enumeration and tends to attenuate over time.

## References

[1] UNAIDS (2020). Global HIV & AIDS statistics—2020 fact sheet.

[2] Frank TD, Carter A, Jahagirdar D (2019). Global, regional, and national incidence, prevalence, and mortality of HIV, 1980–2017, and forecasts to 2030, for 195 countries and territories: a systematic analysis for the Global Burden of Diseases, Injuries, and Risk Factors Study 2017. Lancet HIV.

[3] Vitoria M, Vella S, Ford N (2013). Scaling up antiretroviral therapy in resource-limited settings: adapting guidance to meet the challenges. Curr Opin HIV AIDS.

[4] Pandey A, Galvani AP (2019). The global burden of HIV and prospects for control. Lancet HIV.

[5] World Health Organisation (2016). Progress Report 2016: Prevent HIV, Test and Treat All: WHO Support for Country Impact.

[6] UNAIDS (2021). Understanding fast-track: acelerating action to end the AIDS epidemic by 2030.

[7] UNAIDS 90-90-90: treatment for all.

[8] Ford N, Meintjes G, Calmy A (2018). Managing advanced HIV disease in a public health approach. Clin Infect Dis.

[9] Carmona S, Bor J, Nattey C (2018). Persistent high burden of advanced HIV disease among patients seeking care in South Africa’s national HIV program: data from a nationwide laboratory cohort. Clin Infect Dis.

[10] Bisson GP, Ramchandani R, Miyahara S (2017). Risk factors for early mortality on antiretroviral therapy in advanced HIV-infected adults. AIDS.

[11] Kaplan SR, Oosthuizen C, Stinson K (2017). Contemporary disengagement from antiretroviral therapy in Khayelitsha, South Africa: a cohort study. PLoS Med.

[12] Stinson K, Goemaere E, Coetzee D (2017). Cohort profile: the Khayelitsha antiretroviral programme, Cape Town, South Africa. Int J Epidemiol.

[13] Osler M, Hilderbrand K, Goemaere E (2018). The continuing burden of advanced HIV disease over 10 years of increasing antiretroviral therapy coverage in South Africa. Clin Infect Dis.

[14] Provincial Government of the Western Cape (2015). The Western Cape Consolidated Guidelines for HIV Treatment: Prevention of Mother-to-Child Transmission of HIV (PMTCT), Children, Adolescents and Adults.

[15] World Health Organisation (2004). Treating 3 Million People Living with HIV/AIDS by 2005: Making it Happen: The WHO Strategy.

[16] National Department of Health SA (2015). National Consolidated Guidelines for the Prevention of Mother-To-Child Transmission of HIV (PMTCT) and the Management of HIV in Children, Adolescents, and Adults.

[17] World Health Organisation (2015). Guideline on when to Start Antiretroviral Therapy and on Pre-exposure Prophylaxis for HIV.

[18] National Department of Health SA (2016). Implementation of the Universal Test and Treat Strategy for HIV Positive Patients and Differentiated Care for Stable Patients.

[19] Lambert PC, Royston P (2009). Further development of flexible parametric models for survival analysis. Stata J.

[20] 2019 RsT (2019). RStudio: Integrated Development for R.

[21] Ousley J, Niyibizi AA, Wanjala S (2018). High proportions of patients with advanced HIV are antiretroviral therapy experienced: hospitalization outcomes from 2 sub-Saharan African sites. Clin Infect Dis.

[22] McCreesh N, Andrianakis I, Nsubuga RN (2017). Universal test, treat, and keep: improving ART retention is key in cost-effective HIV control in Uganda. BMC Infect Dis.

[23] Eisinger RW, Dieffenbach CW, Fauci AS (2019). HIV viral load and transmissibility of HIV infection: undetectable equals untransmittable. JAMA.

[24] Hayes RJ, Donnell D, Floyd S (2019). Effect of universal testing and treatment on HIV incidence—HPTN 071 (PopART). N Engl J Med.

[25] Skarbinski J, Rosenberg E, Paz-Bailey G (2015). Human immunodeficiency virus transmission at each step of the care continuum in the United States. JAMA Intern Med.

[26] Shoko C, Chikobvu D (2019). A superiority of viral load over CD4 cell count when predicting mortality in HIV patients on therapy. BMC Infect Dis.

[27] Lamp K, McGovern S, Fong Y (2020). Proportions of CD4 test results indicating advanced HIV disease remain consistently high at primary health care facilities across four high HIV burden countries. PLoS One.

[28] Masaba RO, Herrera N, Siamba S (2023). Advanced HIV disease in Homa Bay County, Kenya: characteristics of newly-diagnosed and antiretroviral therapy-experienced clients. Medicine (Baltimore).

[29] Post FA, Szubert AJ, Prendergast AJ (2018). Causes and timing of mortality and morbidity among late presenters starting antiretroviral therapy in the REALITY trial. Clin Infect Dis.

[30] World Health Organisation (2017). Guidelines for Managing Advanced HIV Disease and Rapid Initiation of Antiretroviral Therapy, July 2017.

[31] Gupta A, Nadkarni G, Yang W-T (2011). Early mortality in adults initiating antiretroviral therapy (ART) in low-and middle-income countries (LMIC): a systematic review and meta-analysis. PLoS One.

[32] Manosuthi W, Charoenpong L, Santiwarangkana C (2021). A retrospective study of survival and risk factors for mortality among people living with HIV who received antiretroviral treatment in a resource-limited setting. AIDS Res Ther.

[33] Nhemachena T, Späth C, Arendse KD (2022). Between empathy and anger: healthcare workers’ perspectives on patient disengagement from antiretroviral treatment in Khayelitsha, South Africa—a qualitative study. BMC Prim Care.

[34] de Bruin M, Oberjé EJM, Viechtbauer W (2017). Effectiveness and cost-effectiveness of a nurse-delivered intervention to improve adherence to treatment for HIV: a pragmatic, multicentre, open-label, randomised clinical trial. Lancet Infect Dis.

[35] Keiser O, Tweya H, Boulle A (2009). Switching to second-line antiretroviral therapy in resource-limited settings: comparison of programmes with and without viral load monitoring. AIDS.

[36] Bray S, Gedeon J, Hadi A (2012). Predictive Value of CD4 Cell Count Nadir on Long-Term Mortality in HIV-Positive Patients in Uganda. HIV AIDS (Auckl).

[37] Siti-Azrin AH, Norsaadah B, Lee SC (2023). Predictors of discontinuation of antiretroviral therapy among HIV-infected adults at Hospital Sungai Buloh: a 10-year retrospective cohort study. Gulhane Med J.

[38] Ehrenkranz P, Rosen S, Boulle A (2021). The revolving door of HIV care: revising the service delivery cascade to achieve the UNAIDS 95-95-95 goals. PLoS Med.

[39] Decroo T, Rasschaert F, Telfer B (2013). Community-based antiretroviral therapy programs can overcome barriers to retention of patients and decongest health services in sub-Saharan Africa: a systematic review. Int Health.

[40] Nachega JB, Adetokunboh O, Uthman OA (2016). Community-based interventions to improve and sustain antiretroviral therapy adherence, retention in HIV care and clinical outcomes in low-and middle-income countries for achieving the UNAIDS 90-90-90 targets. Curr HIV/AIDS Rep.

[41] Fatti G, Ngorima-Mabhena N, Mothibi E (2020). Outcomes of three-versus six-monthly dispensing of antiretroviral treatment (ART) for stable HIV patients in community ART refill groups: a cluster-randomized trial in Zimbabwe. J Acquir Immune Defic Syndr.

[42] Hoffman RM, Moyo C, Balakasi KT (2021). Multimonth dispensing of up to 6 months of antiretroviral therapy in Malawi and Zambia (INTERVAL): a cluster-randomised, non-blinded, non-inferiority trial. Lancet Glob Health.

[43] Bershteyn A, Jamieson L, Kim H-Y (2022). Transmission reduction, health benefits, and upper-bound costs of interventions to improve retention on antiretroviral therapy: a combined analysis of three mathematical models. Lancet Glob Health.

[44] Cornell M, Schomaker M, Garone DB (2012). Gender differences in survival among adult patients starting antiretroviral therapy in South Africa: a multicentre cohort study. PLoS Med.

[45] Cornell M, McIntyre J, Myer L (2011). Men and antiretroviral therapy in Africa: our blind spot. Trop Med Int Health TM IH.

[46] Arnesen R, Moll AP, Shenoi SV (2017). Predictors of loss to follow-up among patients on ART at a rural hospital in KwaZulu-Natal, South Africa. PLoS One.

[47] Prabhu S, Harwell JI, Kumarasamy N (2019). Advanced HIV: diagnosis, treatment, and prevention. Lancet HIV.

[48] Alabi A, Kordy F, Lam R (2020). The complete blood count in children and adolescents with tuberculosis: utility and prevalence of anaemia, lymphopenia and neutrophilia. SN Compr Clin Med.

[49] Hermans SM, Zinyakatira N, Caldwell J (2021). High rates of recurrent tuberculosis disease: a population-level cohort study. Clin Infect Dis.

[50] Sullivan A, Nathavitharana RR (2022). Addressing TB-related mortality in adults living with HIV: a review of the challenges and potential solutions. Ther Adv Infect Dis.

[51] Walker NF, Meintjes G, Wilkinson RJ (2013). HIV-1 and the immune response to TB. Future Virol.

[52] Hsu DC, Kerr SJ, Thongpaeng P (2014). Incomplete restoration of Mycobacterium tuberculosis-specific-CD4 T cell responses despite antiretroviral therapy. J Infect.

[53] Walker AS, Prendergast AJ, Mugyenyi P (2012). Mortality in the year following antiretroviral therapy initiation in HIV-infected adults and children in Uganda and Zimbabwe. Clin Infect Dis.

[54] Low A, Gavriilidis G, Larke N (2016). Incidence of opportunistic infections and the impact of antiretroviral therapy among HIV-infected adults in low-and middle-income countries: a systematic review and meta-analysis. Clin Infect Dis.

[55] WHO (2019). Global Tuberculosis Report 2019: Fact sheet.

[56] Blasich NP, Coetzee LM, Sriruttan C (2022). Retrospective assessment of a national reflex cryptococcal antigen screening program in South Africa through interlaboratory comparison of lateral flow assay results. Lab Med.

[57] Buthelezi SSS (2023). 2023 ART clinical guidelines for the management of HIV in adults, pregnancy and breastfeeding, adolescents, children, infants and neonates. SA Pharm J.

[58] Boulle A, Heekes A, Tiffin N (2019). Data centre profile: the provincial health data centre of the Western Cape Province, South Africa. Int J Popul Data Sci.

[59] Nosyk B, Humphrey L (2022). Highlighting the need for investment and innovation in ART retention interventions. Lancet Glob Health.

