# Supplementary material for: Characteristics of Individuals With Advanced HIV Disease and Risk Factors for Mortality in a Contemporary Cohort in South Africa
Source: J Acquir Immune Defic Syndr. Author manuscript; Available in PMC 2026 Jan 30. (PMC7618700; doi:10.1097/QAI.0000000000003767)

# Addendum A1

As of 2020 Western Cape Consolidated Guidelines for HIV treatment include:

- **CD4 cell counts:** at baseline (diagnosis), and 12 months on ART. CD4 cell count is only repeated if 12 month CD4 is <200cells/mm^3^ in which case it is repeated 6-monthly until > 200 cells/mm^3^; or if a participant has a viral load (VL) > 1000 copies/ml on two consecutive tests in which case it is repeated 6-monthly to monitor for immunological failure.
- **Viral Load (VL):** On first line drug therapy, VL is performed at month 4, month 12 and then annually. If a viral load is > 50 copies/ml it is then repeated within three months to allow for detection of virological failure.
- **Serum Creatinine:** Serum Creatinine (and eGFR as per the MDRD formula for adults > 18) is performed on all individuals initiated on tenofovir, which is included in the vast majority of first-line ART regimens initiated in the Western Cape. Serum Creatinine is repeated for all these individuals at month 1, 4 and 12 and then annually for all individuals while remaining on tenofovir.

Other routine laboratory investigations include a sputum TB GeneXpert test for all symptomatic individuals and a reflex Cryptococcal Antigen (CrAg) test on all individuals with a baseline CD4 cell count < 100cells/mm^3^. Reflex CrAg testing has existed as a South African national screening programme since 2016^60^.

# Addendum A2

| **Prior to 2010**^12,15^ | **April 2010**^12^ | **April 2013**^12^ | **April 2015**^16^ | **September 2016**^17,18^ |
| --- | --- | --- | --- | --- |
| WHO stage IV or CD4 count <200cells/mm^3^ | WHO stage IV or CD4 count <200cells/mm^3^ for all adults  Or CD4 cells count <350cells/mm^3^ for individuals with TB or in pregnancy | WHO stage III or IV or CD4 count <350cells/mm^3^  Or any individual with TB, pregnant or breastfeeding | WHO stage III or IV or CD4 count <500cells/mm^3^ | ART accessible to all |

## Addendum B ^61^

**Table 3:** Types and degrees of evidence used to infer tuberculosis by the PDHC

| **Inferred conditions** | **High certainty (Strong evidence)** | | | |  | | **Moderate** | | | |
| --- | --- | --- | --- | --- | --- | --- | --- | --- | --- | --- |
|  | **Laboratory tests** | **Drugs dispensed** | **Procedure or ICD-10 codes** | **Other evidence** |  | **Laboratory tests** | | **Drugs dispensed** | **Procedure or ICD-10 codes** |  |
| **Tuberculosis** | positive GeneXpert or  line probe assay or  microscopy, culture & microbiology | tuberculosis treatment regimen | tuberculosis  ICD-10 code (A15) | admitted in tuberculosis hospital;  registered in tuberculosis electronic chronic disease management system |  |  | |  |  |  |
| ICD-10 International Statistical Classification of Diseases and Related Health Problems 10th Revision | | | | | | | | | | |

## Addendum C

**Enumeration:** Date of first CD4 count <200 cells/mm^3^ within the study period

**On ART:** Documented collection of ART from a linked pharmacy within 90 days prior to enumeration

**Disengaged:** Documented prior collection of ART from a linked pharmacy but not within 90 days of enumeration

**ART Naïve:** No documented collection of ART from a linked pharmacy prior to enumeration

**Prior ART exposure/ART experience:** Both on ART and disengaged participants

**Enumeration CD4 count:** First documented CD4 count below 200 within the study period

**Enumeration viral load (VL):** Latest viral load done within two months after or one year before enumeration

**NPR linkage:** Documented linkage to the national population registry via a national ID number

**Current TB:** Strong evidence of TB (addendum H) within two months before or after enumeration

**Previous TB:** Strong evidence of TB more than two months before enumeration

**Incident TB:** Strong evidence of TB more than two months after enumeration

**Current cryptococcosis:** Microbiological evidence of *Cryptococcus neoformans* in cerebrospinal fluid (CSF) or plasma/serum within two months before or after enumeration

**Previous cryptococcosis:** Microbiological evidence of *Cryptococcus neoformans* in CSF or plasma/serum more than two months before enumeration

**Incident cryptococcosis:** Microbiological evidence of *Cryptococcus neoformans* in CSF or plasma/serum more than two months after enumeration

## Addendum D

STROBE Statement—Checklist of items that should be included in reports of ***cohort studies***

|  | Item No | Recommendation |  |  |  |
| --- | --- | --- | --- | --- | --- |
| **Title and abstract** | 1 | (*a*) Indicate the study’s design with a commonly used term in the title or the abstract |  | Y |  |
|  |  | (*b*) Provide in the abstract an informative and balanced summary of what was done and what was found |  | Y |  |
| Introduction | | |  |  |  |
| Background/rationale | 2 | Explain the scientific background and rationale for the investigation being reported |  | Y |  |
| Objectives | 3 | State specific objectives, including any prespecified hypotheses |  | Y |  |
| Methods | | |  |  |  |
| Study design | 4 | Present key elements of study design early in the paper |  | Y |  |
| Setting | 5 | Describe the setting, locations, and relevant dates, including periods of recruitment, exposure, follow-up, and data collection |  | Y |  |
| Participants | 6 | (*a*) Give the eligibility criteria, and the sources and methods of selection of participants. Describe methods of follow-up |  | Y |  |
|  |  | (*b*) For matched studies, give matching criteria and number of exposed and unexposed |  | NA |  |
| Variables | 7 | Clearly define all outcomes, exposures, predictors, potential confounders, and effect modifiers. Give diagnostic criteria, if applicable |  | Y |  |
| Data sources/ measurement | 8* | For each variable of interest, give sources of data and details of methods of assessment (measurement). Describe comparability of assessment methods if there is more than one group |  | Y |  |
| Bias | 9 | Describe any efforts to address potential sources of bias |  | Y |  |
| Study size | 10 | Explain how the study size was arrived at |  | Y |  |
| Quantitative variables | 11 | Explain how quantitative variables were handled in the analyses. If applicable, describe which groupings were chosen and why |  | Y |  |
| Statistical methods | 12 | (*a*) Describe all statistical methods, including those used to control for confounding |  | Y |  |
|  |  | (*b*) Describe any methods used to examine subgroups and interactions |  | Y |  |
|  |  | (*c*) Explain how missing data were addressed |  | Y |  |
|  |  | (*d*) If applicable, explain how loss to follow-up was addressed |  | Y |  |
|  |  | (*e*) Describe any sensitivity analyses |  | Y |  |
| Results | | |  |  |  |
| Participants | 13* | (a) Report numbers of individuals at each stage of study—eg numbers potentially eligible, examined for eligibility, confirmed eligible, included in the study, completing follow-up, and analysed |  | Y |  |
|  |  | (b) Give reasons for non-participation at each stage |  | Y |  |
|  |  | (c) Consider use of a flow diagram |  | Y |  |
| Descriptive data | 14* | (a) Give characteristics of study participants (eg demographic, clinical, social) and information on exposures and potential confounders |  | Y |  |
|  |  | (b) Indicate number of participants with missing data for each variable of interest |  | Y |  |
|  |  | (c) Summarise follow-up time (eg, average and total amount) |  | Y |  |
| Outcome data | 15* | Report numbers of outcome events or summary measures over time |  | Y |  |
| Main results | 16 | (*a*) Give unadjusted estimates and, if applicable, confounder-adjusted estimates and their precision (eg, 95% confidence interval). Make clear which confounders were adjusted for and why they were included |  | Y |  |
|  |  | (*b*) Report category boundaries when continuous variables were categorized |  | Y |  |
|  |  | (*c*) If relevant, consider translating estimates of relative risk into absolute risk for a meaningful time period |  | Y |  |
| Other analyses | 17 | Report other analyses done—eg analyses of subgroups and interactions, and sensitivity analyses |  | Y |  |
| Discussion | | |  |  |  |
| Key results | 18 | Summarise key results with reference to study objectives |  | Y |  |
| Limitations | 19 | Discuss limitations of the study, taking into account sources of potential bias or imprecision. Discuss both direction and magnitude of any potential bias |  | Y |  |
| Interpretation | 20 | Give a cautious overall interpretation of results considering objectives, limitations, multiplicity of analyses, results from similar studies, and other relevant evidence |  | Y |  |
| Generalisability | 21 | Discuss the generalisability (external validity) of the study results |  | Y |  |
| Other information | | |  |  |  |
| Funding | 22 | Give the source of funding and the role of the funders for the present study and, if applicable, for the original study on which the present article is based |  | Y |  |

*Give information separately for exposed and unexposed groups.

**Note:** An Explanation and Elaboration article discusses each checklist item and gives methodological background and published examples of transparent reporting. The STROBE checklist is best used in conjunction with this article (freely available on the Web sites of PLoS Medicine at http://www.plosmedicine.org/, Annals of Internal Medicine at http://www.annals.org/, and Epidemiology at http://www.epidem.com/). Information on the STROBE Initiative is available at http://www.strobe-statement.org.

## Addendum E

**Table 4:** Characteristics of the cohort stratified by NPR linkage

|  | Unlinked | Linked | p |
| --- | --- | --- | --- |
| n | 2192 | 11152 |  |
| Mortality (Died) (%) | 0 (0.0) | 1349 (12.1) | <0.001 |
| NPR Linkage (Linked) (%) | 0 (0.0) | 11152 (100.0) | <0.001 |
| Sex (Male) (%) | 1024 (46.7) | 4704 (42.2) | <0.001 |
| Age > 40 (%) | 863 (39.4) | 3640 (32.6) | <0.001 |
| Age (mean (SD)) | 38.37 (9.70) | 36.83 (9.38) | <0.001 |
| Enumeration CD4 (median [IQR]) | 107.00 [56.00, 157.25] | 111.00 [58.00, 158.00] | 0.400 |
| Enumeration CD4 Categorical (%) |  |  | 0.268 |
| 101 - 199 | 1176 (53.6) | 6192 (55.5) |  |
| 51 - 100 | 520 (23.7) | 2553 (22.9) |  |
| 0 - 50 | 496 (22.6) | 2407 (21.6) |  |
| Time in Cohort (median [IQR]) | 973.50 [578.00, 1308.00] | 874.00 [469.75, 1265.00] | <0.001 |
| Viral Load at Enumeration (%) |  |  | 0.031 |
| < 100 | 211 (29.6) | 1100 (33.1) |  |
| 100 -1000 | 66 (9.2) | 365 (11.0) |  |
| > 1000 | 437 (61.2) | 1855 (55.9) |  |
| Enumeration VL Not Done (%) | 1478 (67.4) | 7832 (70.2) | 0.010 |
| ART (%) |  |  | 0.004 |
| On ART | 483 (22.0) | 2148 (19.3) |  |
| Disengaged | 813 (37.1) | 4467 (40.1) |  |
| ART Naive | 896 (40.9) | 4537 (40.7) |  |
| ART Naive + Subsequently Initiated (%) | 833 (93.0) | 4351 (95.9) | <0.001 |
| Time to Initiation (median [IQR]) | 14.00 [2.00, 28.00] | 10.00 [0.00, 28.00] | 0.089 |
| Disengaged + Re-initiated (%) | 736 (90.5) | 4117 (92.2) | 0.133 |
| Time to Re-initiation (median [IQR]) | 5.00 [0.00, 21.00] | 7.00 [0.00, 26.00] | 0.033 |
| Enumerated in Hospital (%) | 393 (17.9) | 1670 (15.0) | 0.001 |
| Current TB (PHDC) (%) | 611 (27.9) | 2789 (25.0) | 0.005 |
| Previous TB (PHDC) (%) | 831 (37.9) | 3563 (31.9) | <0.001 |
| Incident TB (PHDC) (%) | 351 (16.0) | 1861 (16.7) | 0.456 |
| Current Cryptococcosis (%) | 40 (1.8) | 185 (1.7) | 0.645 |
| Previous Cryptococcosis (%) | 27 (1.2) | 92 (0.8) | 0.084 |
| Incident Cryptococcosis (%) | 36 (1.6) | 176 (1.6) | 0.900 |
| Reflex LFA Positive (%) | 58 (100.0) | 271 (100.0) |  |
| Diabetic (%) | 65 (3.0) | 283 (2.5) | 0.282 |
| Hypertension (%) | 247 (11.3) | 945 (8.5) | <0.001 |
| Chronic Kidney Disease (%) | 47 (2.1) | 215 (1.9) | 0.560 |

## Addendum F


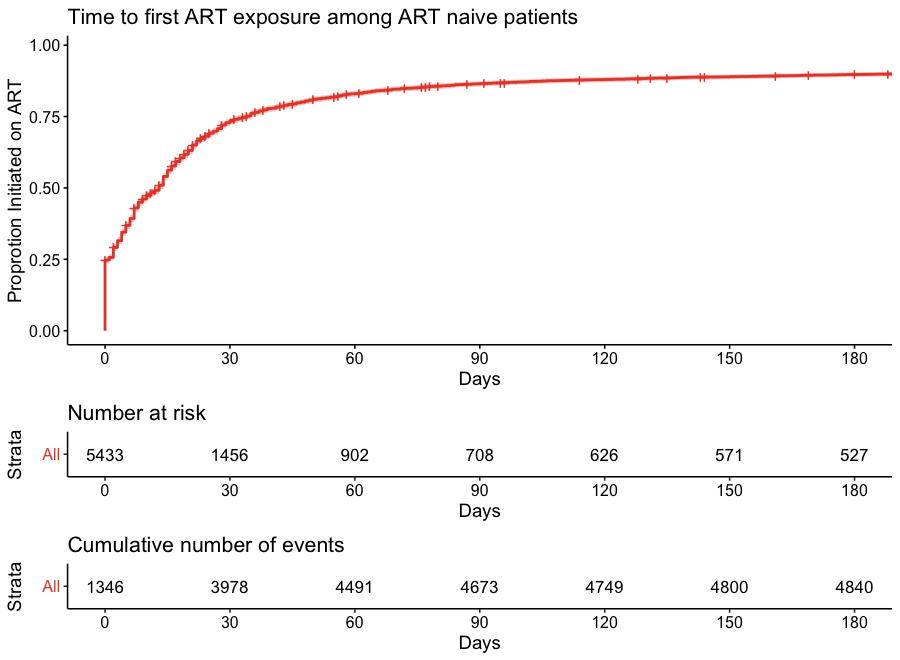

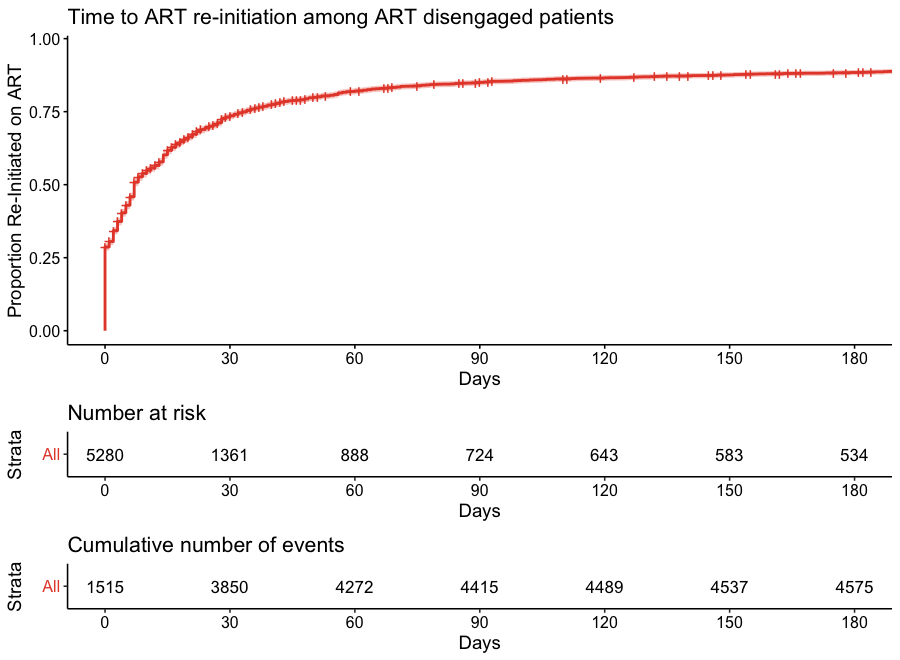


**Figure 3:** Kaplan Meier curves of time to ART re-initiation (A) or initiation (B) amoung ART disengaged (A) and naïve (B) participants and censored at 180 days.

## Addendum G


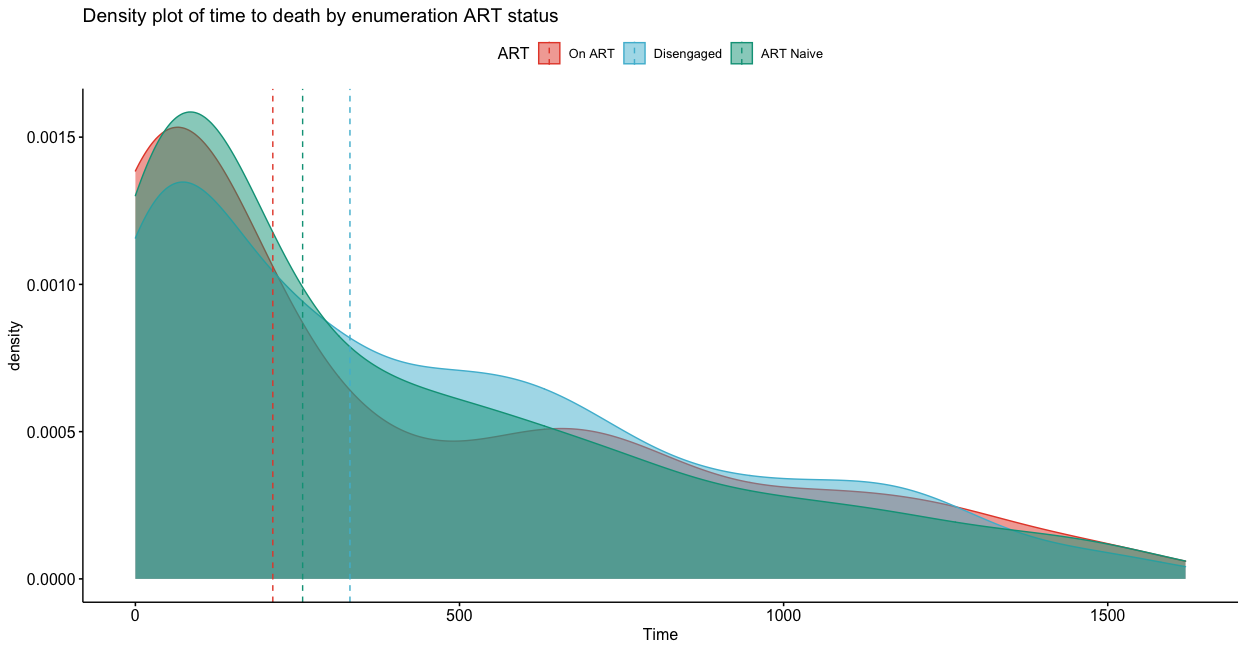


**Figure 5:** Density plot of time to death by enumeration ART status

## Addendum H

Sensitivty analysis comparing the Kaplan-Meier curves before (above) and after (below) changing the definition of ART naïve to include those who first collected ART within 3 months (90 days) prior to enrolment.


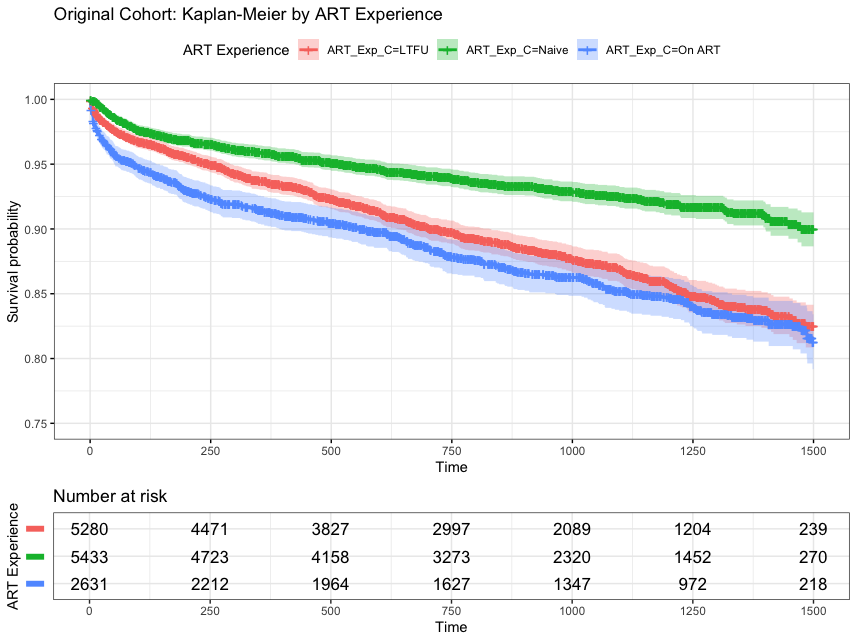


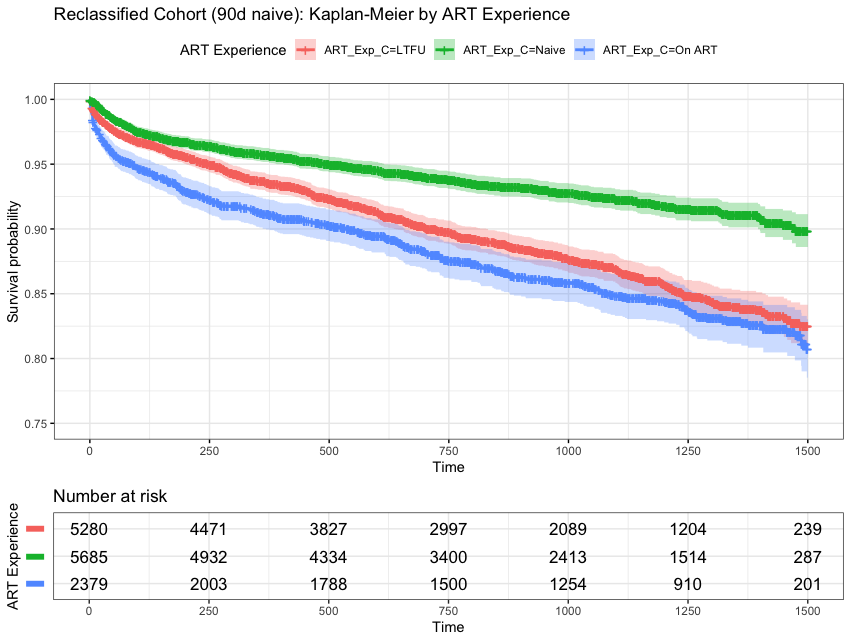

Supplement: Supplemental Digital Content [file EMS212255-supplement-Supplemental_Digital_Content.docx]
